# Scoping review of the use of multimorbidity variables in cardiovascular disease risk prediction

**DOI:** 10.1186/s12889-025-22169-6

**Published:** 2025-03-17

**Authors:** Emma Church, Katrina Poppe, Susan Wells

**Affiliations:** University of Auckland, Auckland, New Zealand

**Keywords:** Cardiovascular diseases, Risk prediction, CVD risk, Population health, Multimorbidity, Comorbidity, Charlson comorbidity index

## Abstract

**Background:**

Cardiovascular disease (CVD) is a leading cause of morbidity and mortality globally. Many countries use pooled cohort equations or similar risk prediction models to assess atherosclerotic CVD risk to guide preventive measures. There is evidence that clinical CVD risk prediction equations are less accurate for adults with higher levels of multimorbidity (the co-occurrence of multiple long-term conditions). Operating within a single disease paradigm may not be appropriate for adults with multimorbidity who may be at higher risk of both CVD and non-CVD death. This scoping review was conducted to gather evidence on the inclusion of multimorbidity measures in CVD risk models to assess their methodology and identify evidence gaps in the literature.

**Methods:**

The review covers literature from 1 January 2012 to 23 September 2022, using the Arksey and O’Malley framework. We searched MEDLINE, Embase, and Cochrane databases published during this period and followed the Preferred Reporting Items for Systematic Reviews and Meta-Analyses extension for Scoping Reviews (PRISMA-ScR) reporting guidelines.

**Results:**

This review identified fourteen studies reporting multivariable prognostic CVD models that included a multimorbidity variable. Of these, four studies specifically looked at the added benefit of a multimorbidity variable in a CVD risk model. Only one of these studies was conducted in a primary prevention cohort (i.e., people were free of CVD at baseline). This scoping review revealed several primary evidence gaps, notably the limited literature on the topic, the model performance in ethnic subpopulations, and the comparative assessment of alternative multimorbidity variables beyond the Charlson Comorbidity Index.

**Conclusions:**

Few studies have assessed the impact of incorporating multimorbidity indices in primary and secondary prevention cohorts. Future research is needed to evaluate the incremental value of multimorbidity indices in cardiovascular disease risk prediction models to inform risk stratification and management strategies in people with multimorbidity.

**Supplementary Information:**

The online version contains supplementary material available at 10.1186/s12889-025-22169-6.

## Background

Globally, cardiovascular diseases (CVDs), primarily ischemic heart disease and cerebrovascular disease, are the leading cause of mortality and a significant contributor to disability [1]. The co-occurrence of other long-term health conditions or multimorbidity with CVD is increasing [2]. In a large primary care cohort in the United Kingdom (UK), Tran et al. found that while incident cases of nonfatal CVD between 2000 and 2014 decreased by 34%, the proportion of CVD patients with comorbidities increased (e.g., 6% of CVD patients had five or more comorbidities in 2000, increasing to 24% in 2014 in age/sex standardised models) [3]. There is evidence that measures of multimorbidity are independently associated with a range of CVD outcomes, including in adults without prior CVD [4, 5] and adults with a clinical history of CVD [6–8]. In addition, Tonelli et al. found several non-cardiovascular comorbidities associated with higher rates of myocardial infarction (or “discordant” comorbidity) in a chronic kidney disease cohort [9]. Therefore, a single disease paradigm may not be appropriate for adults with multimorbidity, as CVD risk in such individuals may be influenced by both the higher risk of non-CVD death and the increased CVD risk from other baseline comorbidities.

Assessing CVD risk is important for targeted CVD primary prevention, balancing benefits/harms in CVD risk management, and aiding general practitioners and their patients in making informed decisions. Many clinical CVD risk tools are available to measure the probability of developing CVD [10]. Nguyen assessed the performance of American College of Cardiology/American Heart Association recommended Pooled Cohort Equations for 10-year risk of atherosclerotic CVD events in older adults who were free of CVD at baseline in the United States (US) and found that calibration and discriminatory ability of the pooled cohort equation varied across multimorbidity subgroups (e.g., overprediction of risk in the cardiometabolic classes and underprediction of risk in the low cognition and musculoskeletal-lung depression classes) [11]. Internationally, the UK QRISK3 is the only CVD risk equation to include a wide selection of non-CVD comorbidities [12]. Derived from a large United Kingdom primary care electronic health record dataset, QRISK3 predicts 10-year fatal and nonfatal CVD events. It includes over 20 predictor variables for CVD, including diabetes, treated hypertension, atrial fibrillation, rheumatoid arthritis, erectile dysfunction, chronic kidney disease stages, severe mental illness, treatment with atypical antipsychotic drugs, HIV/AIDS, and systemic lupus erythematosus. However, QRISK3 may be less effective at identifying individuals at higher risk due to the cumulative effect of multiple conditions. Livingstone et al. evaluated the QRISK3 10-year predicted CVD risk with the observed risk in multimorbidity subgroups in a primary care cohort and found that the QRISK3 overpredicts CVD risk in people with high levels of multimorbidity [13]. 

There is no singular method for quantifying multimorbidity in health research to study specific multimorbidity-related outcomes. Numerous methods have been developed and can be categorised into four main groups: (1) simple counts of chronic diseases or medication groups, (2) weighted methods (conditions or classes of medication weighted according to their relative impact on a specific outcome of interest), (3) organ or system-based approaches (assessing impacts of chronic conditions on body systems), and (4) other miscellaneous approaches [14]. This scoping review aimed to identify studies that included multimorbidity measures in CVD risk prediction models. Specifically, we sought to describe the populations or cohorts within the included studies, identify the characteristics of the multimorbidity instruments, assess the contribution of the multimorbidity variables to the performance of the CVD risk models, and note any author-described limitations.

## Methods

A scoping review was selected to outline what is known about this topic, identify evidence gaps, and inform further research [15]. This review followed the Arksey and O’Malley scoping review methodological framework [16–19], which comprises five steps: (i) identifying the research question, (ii) identifying relevant studies, (iii) selecting eligible studies, (iv) charting the data, and (v) collating and summarising the results. This review was reported according to the Preferred Reporting Items for Systematic Reviews and Meta-Analyses extension for Scoping Reviews [PRISMA-ScR] statement (Checklist in Additional File 1). A research protocol was developed a-priori with minor changes added after peer review, a preliminary literature search, and advice from a medical librarian. The final version of the protocol is available upon request from the corresponding author.

### Search strategy

The search strategy was developed with support from a medical librarian across three medical databases (Medline, Embase, and Cochrane). The search strategy for each database is presented in Additional File 2. Searches conducted on 23 September 2022 covered articles from 1 January 2012 onward to align with contemporary developments in multimorbidity measurement and CVD risk prediction methodologies. We also manually searched reference lists for additional relevant articles.

The results from each database and manual searches were exported to a single library in the RefWorks^®^ reference manager. Duplicate studies were identified and removed.

The primary reviewer (EC) conducted title and abstract screening using the inclusion and exclusion criteria to determine the eligibility of the selected and identified studies for review and subsequently conducted article and full-text screening of all eligible articles. When inclusion was unclear, an agreement was reached after discussion with the second and third authors.

### Inclusion and exclusion criteria

Articles were included if they (a) were published from 1 January 2012 until 23 September 2022, (b) were available in English, (c) were primary quantitative studies, (d) were conducted in any adult population (community, hospital, primary care, any country, presence of index condition), (e) included a multimorbidity variable/instrument (e.g., disease count, index, drug count, etc.) in an atherosclerotic CVD risk model, (f) used model/s predictors collected at baseline or at the time of recruitment into the cohort, and (g) evaluated the prognostic performance of the CVD risk model. Polypharmacy was included as a potential multimorbidity variable as it is essentially a drug count with a threshold applied.

Articles were excluded if they (a) used postsurgical or in-hospital complications outcomes, (b) used pediatric populations (< 18 years old), (c) used only composite CVD outcomes that included all-cause mortality or other non-CVD outcomes, (d) only used prognostic scores for an index condition (e.g., a heart failure prognostic score) rather than a multimorbidity measure, (e) where a full-text article could not be obtained (except in the case of conference abstracts that provided sufficient information) or (f) included dynamic risk modelling (where candidate predictors vary over time) or repeated measures modelling.

### Data charting, synthesis and reporting

A data charting form was developed by EC and used to capture relevant information from each included study. All authors agreed upon minor changes before finalising the form (Additional File 3). Extracted information included study design and methodology, study population and setting, multimorbidity measures in the CVD risk model, reference model variables, limitations, and conclusions. Data from all the included studies were extracted by the primary reviewer (EC) and reviewed by KP and SW.

The extracted data were summarised around the following themes: the populations studied, types and characteristics of multimorbidity variables used in CVD risk models, whether the incremental value of the multimorbidity variable to existing predictors was able to be assessed, how the performance of the models was evaluated, and any limitations reported.

## Results

**Fig. 1:**
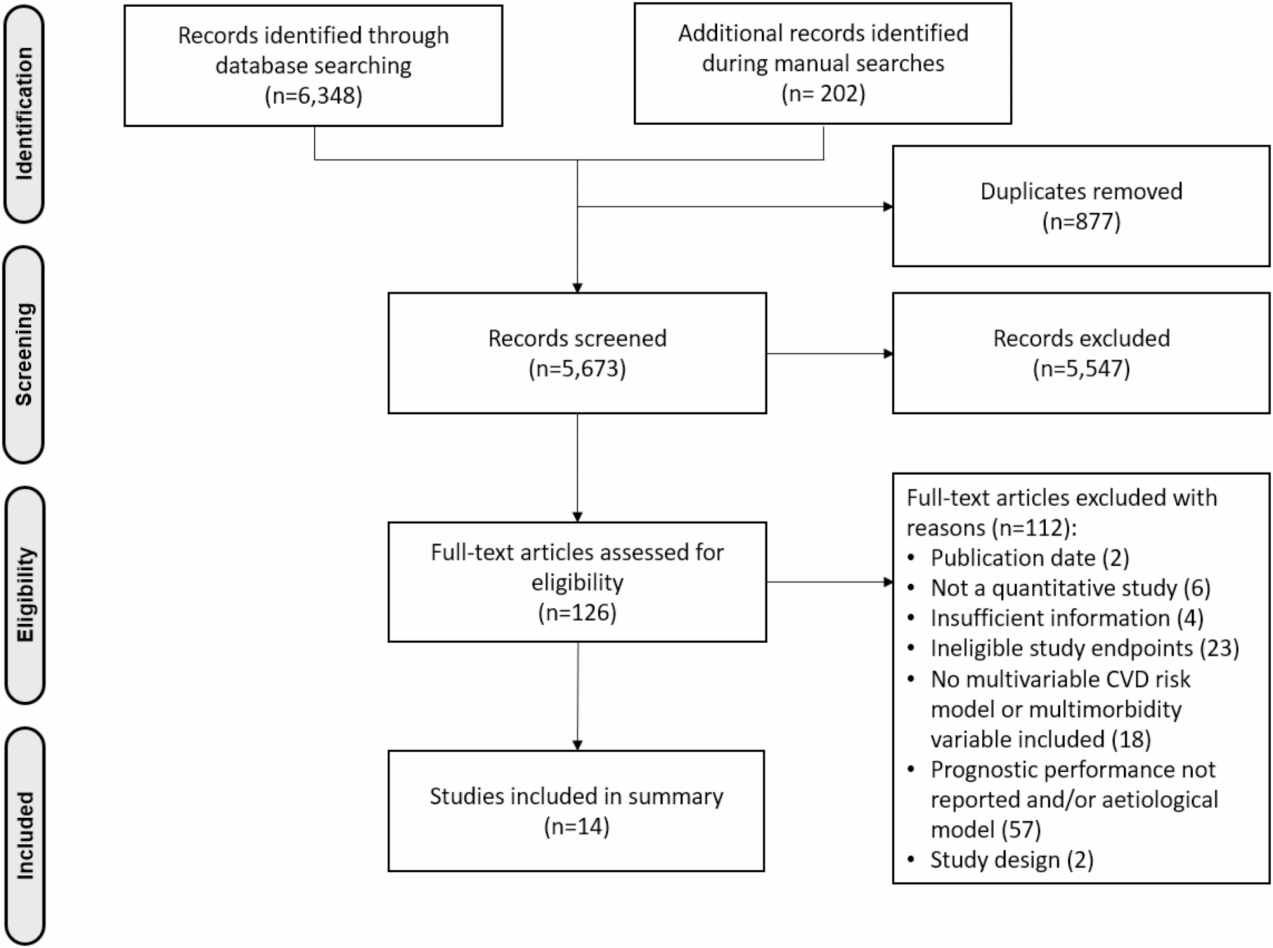
PRISMA diagram

The database search yielded 6,348 articles, and 202 were identified from other sources. After removing 877 duplicates, we screened the titles and abstracts of 5,673 articles and excluded 5,547 articles. We conducted a full-text screening of the remaining 126 articles, excluding 112 for reasons outlined in the PRISMA diagram (Fig. 1). We extracted data from the remaining 14 articles.

### Study and population characteristics

The study characteristics, multimorbidity measures and methods used by the 14 studies are presented in Table 1. Out of the 14 primary quantitative studies, 10 were journal articles [20, 21, 26, 27, 29–33] and four were conference abstracts [22–24, 28]. Of those studies that reported the cohort type, three were primary prevention cohorts (patients did not have clinically evident CVD at baseline) [27, 30–32], five were secondary prevention cohorts (patients had CVD at baseline) [21, 24, 26, 28] and four were mixed cohorts (patients were included regardless of whether they had clinically relevant CVD at baseline) [20, 29, 33]. The populations studied included two primary care cohorts [30, 31], two populations of coronary artery disease patients [21, 26], three heart failure cohorts [24, 28], two atrial fibrillation cohorts [22, 23] and one population each of colorectal cancer survivors [27], sepsis survivors [32], medical claims participants [29], and nonsurgical patients [20]. Four studies had fewer than 1,000 participants [20, 25, 26, 28] and in five of the included studies, the study population was split into a derivation/training cohort and a validation cohort (all separate holdout cohorts) [27, 29, 30, 32, 33]. 

**Table 1:** A comparison of study characteristics and definitions of Multimorbidity used in the 14 articles

Author/year	Patients/setting	Prevention cohort type	CVD Outcome/s (number of events)	MM measure (competing risk model)	AdjHR of MM measure	Metrics used for performance	Contribution of MM assessed
Bahrmann et al. (2019) [20]	307 non-surgical patients ≥ 68 years presenting to the Emergency department. Germany.	Mixed	1-year CVD mortality (30)	CCI original version	1.16 (1.02–1.31) in fully adjusted model	Model fit (AIC).	Lowest AIC 317.7 for model 3 (assessing the addition of CCI to Barthel index, age, and sex).
Erickson et al. (2014) [21]	1,202 Patients ≥ 18 years hospitalised with unstable angina or acute myocardial infarction. 83.4% White. United States (US).	Secondary	Occurrence of secondary events within 6 months (317)	CCI D’Hoore version	adjOR 1.09 (1.02–1.16) in combined model	Calibration: Hosmer–Lemeshow test, Discrimination: c-statistic, Likelihood ratio test.	Likelihood ratio test indicated that adding the CCI to a model containing the GRPI improved model predictive ability (chi-square value 16.031).
Fauchier et al. (2019)* [22]	8,962 consecutive patients with atrial fibrillation. France.	Unspecified	CVD mortality. Mean follow-up of 929 days, SD 1082 days (1,294 patients died, and 54% were documented cardiovascular deaths)	CCI version unspecified in abstract	NA	Discrimination: AUC.	No
Fauchier et al. (2020)* [23]	2,641,626 patients with atrial fibrillation. France.	Unspecified	Cardiovascular death. Mean follow-up 2.0 years, SD 2.3 years (670,541 died, 30.3% cardiovascular deaths)	CCI version unspecified in abstract	NA	Discrimination: AUC.	No
Fauchier et al. (2022)* [24]	371,848 consecutive patients hospitalised with heart failure. France.	Secondary	CVD death. Mean follow-up 4.0 years, SD 2.8 years (220,774 patients died and 31.3% CVD deaths)	CCI unspecified version in abstract	NA	Discrimination: AUC.	No
Gelow et al. (2015) [25]	246 heart failure patients. Mean age 56.6 years, SD 13.1 years. 88.4% White. US.	Secondary	Fatal and non-fatal CVD events within 6 months (77)	CCI unspecified version	NA	Discrimination: Harrell’s c-statistic.	No
Haji et al. (2022) [26]	334 coronary artery disease patients ≥ 45 years screened during cardiac admission. Australia.	Secondary	3-year heart failure-related hospitalisations (50)	CCI unspecified version (Competing risk model)	SHR 1.64 (1.25–2.15) in fully adjusted model (“Model 5”)	Discrimination: AUC.	No
Jeong et al. (2022) [27]	8,666 newly diagnosed patients with colorectal cancer who survived at least 5 years (training cohort = 4,709, validation cohort = 3,957). Median age 60 years, IQR 53–67 years. South Korea.	Primary	1-to-5-year CVD hospitalisations and ischaemic heart disease hospitalisations (295 CVD events and 127 ischaemic heart disease events in validation cohort)	CCI unspecified version	1.035 (1.003–1.067) in total CVD model and 1.054 (1.007–1.102) IHD model for the training cohort	Discrimination: AUC, Calibration: plots assessed.	No
Kayama et al. (2018)* [28]	376 acute decompensated heart failure patients. Japan.	Secondary	Composite of cardiac death and heart failure hospitalisation. Mean follow-up period 2.0 years, SD 1.4 years (137)	Age-adjusted Charlson comorbidity index (ACCI).	ACCI 0–4: Ref ACCI ≥ 7: 2.69 (1.65–4.36)	Discrimination: AUC.	No
Lip et al. (2022) [29]	3,435,224 people (67% in the training cohort and 33% in the validation cohort), from two health plans. Mean age 49.7 years, SD 15.3 years. US.	Mixed	The first incidence of stroke. Follow-up period not specified. (53,010 in total cohort)	Multimorbid index	NA	Discrimination: c-statistic, Calibration: plots assessed, Decision curve analysis for clinical utility.	No
Livingstone et al. (2022) [30]	2,904,773 primary care patients (derivation cohort and validation cohorts: *n* = 989,732 and 494,865 women, *n* = 946,784 and 473,392 men) who were free of CVD (age 25–84 years). 91.8% White. UK.	Primary	10-year fatal and non-fatal first CVD event (14,150 for women and 17,689 for men in derivation cohort)	modified CCI– using an adapted version for Read codes (Competing risk model)	SHR in derivation cohort. CCI 0: Ref CCI ≥ 3: Women 1.18 (0.94–1.49), Men 1.17 (0.93–1.46)	Model fit (AIC), Calibration: plots assessed, Discrimination Harrell’s c-statistic, patient reclassification and estimated NNT.	Assessed incremental value within sex-stratified competing risks models with QRISK3 predictors, improving calibration (including by age and CCI subgroups) and patient reclassification.
van Bussel et al. (2019) [31]	1,811 primary care patients aged 70-78 years. 97.8% Caucasian. Netherlands.	Primary	5-year risk of combined CVD morbidity and mortality (277)	Polypharmacy (Binary) (Competing risk model)	1.41 (1.08-1.83) in the Cox-PH model and SHR 1.40 (1.08–1.82) in the Fine-Gray models	Calibration plots assessed, Discrimination IPCW c-statistic.	No
Walkey et al. (2022) [32]	39,590 KPNC and 16,388 IH sepsis survivors (two cohorts), median ages 70.0 years (IQR 60–81) years and 66 years (IQR 56–76), respectively. 62.7% White. US.	Mixed (although it excluded those with a CVD history in five years before baseline)	1-year atherosclerotic CVD outcomes (996 at KP and 192 at IH).	CCI unspecified version, as a presepsis risk factor. (Competing risk model)	1.05 (0.96–1.14) for CCI in the KPNC model for combined CVD outcomes	Calibration plots assessed, Discrimination c-statistic.	No
Zhan et al. (2020) [33]	428,251 COPD patients (training cohort *n* = 214,126 and validation cohort *n* = 214,125) mean age 67 years, SD 13 years. Taiwan.	Mixed	1-year CVD hospitalisation (16,071 in the training dataset, 15,723 in the validation dataset) and cerebrovascular hospitalisation (7,337 in the training cohort, 7,306 in the validation cohort)	Charlson Index (Deyo), the updated CDS, and the PBDI.	NA	Calibration plots assessed, Discrimination c-statistic, Patient reclassification and NRI.	Compared to a reference group with age and sex only with different models for each index showing improved discrimination and reclassification ability of the comorbidity models.

*Conference abstract

Abbreviations: MM, multimorbidity; adjHR, adjusted hazard ratio; adjOR, adjusted odds ratio; CCI, Charlson Comorbidity Index; GRPI, Grace Risk Prediction Index; AIC, Akaike Information Criterion; AUC, Area under the Receiver Operating Characteristic (ROC) Curve; IQR, Interquartile range; SD, standard deviation; SHR, Subdistribution hazard ratio; NNT, number needed to treat; IPCW, Inverse Probability of Censoring Weighting; KPNC, Kaiser Permanente Northern California; IH, Intermountain Healthcare; COPD, Chronic Obstructive Pulmonary Disease; CDS, Chronic Disease Score; PBDI, Pharmacy-Based Disease Index; NRI, Net Reclassification Improvement; US, United States

Of the 14 included studies, 11 were conducted in countries with predominantly White/European populations, including Germany [20], the US [21, 25, 29, 32], UK [30], France [22–24], Australia [26] and the Netherlands [31]. Five of these studies report ethnicity data for White participants (ranging from 62.7–97.8%) [21, 25, 30–32]. Only one study provided a detailed breakdown of ethnic groups beyond White participants [32]. The remaining three studies, conducted in South Korea, Taiwan, and Japan, did not report ethnicity data [27, 28, 33]. 

Of the dependent variables relevant for this scoping review, four studies predicted CVD hospitalisations/procedures [21, 27, 32, 33], four studies predicted CVD mortality [20, 22–24], three studies predicted fatal and nonfatal CVD outcomes [25, 30, 31], two studies predicted heart failure-related hospitalisations (one of which included cardiac death as a composite outcome) [26, 28], and one study predicted stroke hospitalisations [29]. In six studies, the CVD outcomes were secondary or additional outcomes, and the primary outcome was all-cause mortality [20–24, 33]. Time horizons varied from 6 months to 10 years.

### Multimorbidity variables

The most commonly used multimorbidity variable was the Charlson Comorbidity Index (CCI), included in 12 studies with a range of 14 to 19 disease categories (depending on the version used or modifications made) [20–28, 30, 32, 33]. Of these, five studies reported the version of the CCI used: the Original CCI [20], the Deyo version of the CCI [33], the age-adjusted CCI [28], the D’Hoore version of the CCI [21] and a version of the CCI adapted for Read codes [30]. These versions of the CCI weight the included conditions according to their relative impact on one-year mortality. Of the seven studies that did not report which version of the CCI, three were conference abstracts; therefore, details were limited.

Zhan et al. compared the CCI to other multimorbidity summary measures, including the updated Chronic Disease Score with 29 disease categories (pharmaceutical-based) and the Pharmacy-based Comorbidity Index with 37 drug categories [33]. Zhan et al. also compared the CCI to the original Elixhauser comorbidity measure, which included 30 indicators in the CVD risk model indicating the presence or absence of the 30 comorbidities, but as this was not a single summary measure, it was excluded. Many papers compared the CCI to other types of risk scores, including three studies using the CHA₂DS₂-VASc score (used for atrial fibrillation stroke risk) [22–24], two frailty indices (claims-based frailty index and hospital frailty risk score) [22–24], the AHEAD heart failure prognostic score [28], the Barthel index (an ordinal scale to measure performance in activities in daily living) [20] and the Grace Risk Prediction Index (designed to predict all-cause mortality after acute coronary syndrome[ACS]) [21]. 

Other multimorbidity-related variables used in the two remaining studies included polypharmacy by van Bussel et al. as a binary variable in a CVD risk prediction model for older people (defined as the chronic use of drugs from ≥ 5 Anatomical Therapeutic Chemical Classification groups) [31]. Van Bussel also conducted sensitivity analyses excluding cardiovascular medications from the definition of polypharmacy. Last, Lip et al. developed a new “Multimorbid” index to predict stroke in patients with and without atrial fibrillation, which is an expanded version of the CHA₂DS₂-VASc score incorporating non-cardiovascular comorbidity beyond traditional CVD risk factors, including 22 disease categories or risk factors and gender, age group and Medicare status [29]. The conditions were weighted according to their influence on stroke risk, and the index was compared to the CHADS2 and CHA₂DS₂-VASc scores (in a population with and without atrial fibrillation).

Data sources used to populate multimorbidity variables varied and included ICD-coded hospitalisation or medical claims [23, 24, 27, 29, 32, 33], clinical trials [26, 31], clinical assessment/history [20], medical note review [25], primary care (Read codes) [30], and an ACS registry (physician entered data) [21]. Two studies did not specify the data sources (both conference abstracts) [22, 28]. 

### Added benefit of including multimorbidity variables in CVD risk models

Four of the fourteen studies assessed the added incremental value of the multimorbidity variable to existing predictors [20, 21, 30, 33]. There was variation in the extent to which they evaluated the model’s prognostic performance. Using a primary prevention cohort (*n* = 989,732 women and 946,784 men in the derivation cohort), Livingstone et al. incorporated the modified CCI into a competing risk CVD model (CRISK-CCI), including the same predictors as QRISK3 (an externally validated prediction tool), to improve the prediction of the competing risk of non-CVD death (as CCI has been externally validated to predict mortality) [30]. The performance of CRISK and CRISK-CCI (competing risk model without and with CCI added) were compared to QRISK3 (standard time to event model) in separate models for men and women in the validation dataset by examining discrimination and calibration of all models (including by subgroups of age and CCI level). Discrimination of the CRISK-CCI was excellent and similar to the CRISK and QRISK3 (for women, c-statistic = 0.863/0.864/0.863 respectively; for men, 0.833/0.819/0.832 respectively). The addition of the CCI score to the CRISK-CCI improved calibration by reducing overprediction at higher levels of predicted risk in women and reducing underprediction in men compared to the CRISK model. For the CRISK-CCI and QRISK3, reclassification of risk was also assessed with respect to treatment thresholds in the UK, demonstrating differences in the type of patients recommended for treatment of CVD risk around these thresholds. Compared with QRISK3, CRISK-CCI recommended fewer people for treatment with a lower estimated number needed to treat to prevent a CVD event.

Using a cohort of patients admitted with ACS (*n* = 1,202), Erickson et al. compared the discrimination and calibration of the CCI to the Grace Risk Prediction Index (GRPI) for several outcomes, including the occurrence of secondary events or procedures for patients with an ACS presentation [21]. The GRPI is used to predict death and death or myocardial infarction six months after ACS presentation [34]. A likelihood ratio test was used to assess the incremental value of incorporating the D’Hoore version of CCI into a model containing GRPI. The test, which assesses the improvement in overall model fit, indicates that adding the D’Hoore CCI significantly improved the model’s ability to capture the observed data (chi-square 16.03, *p* < 0.001).

Using a cohort of chronic obstructive pulmonary disease (COPD) patients (*n* = 428,251), including people who had a prior history of CVD and people without, Zhan et al. compared the prognostic performance (calibration, discrimination and net risk reclassification) of the Deyo version of the CCI, the updated Chronic Disease Score and the Pharmacy-based Comorbidity Index in Taiwan for all-cause mortality and all-cause hospitalisations compared to a reference model with age and sex [33]. Zhan et al. also examined cause-specific outcomes, including cardiovascular and cerebrovascular disease hospitalisations. For cardiovascular hospitalisations, the study found that the Pharmacy-based Comorbidity Index (weights for medication classes derived in the Taiwanese general population) had greater discrimination and reclassification abilities in the validation cohort than the Deyo version of the CCI and the updated Chronic Disease Score.

Last, Bahrmann et al. assessed the incremental value of the original CCI in models containing the Barthel Index, age and sex on prediction of mortality, CVD mortality and rehospitalisation one year after discharge in a cohort of nonsurgical patients (*n* = 307), including patients with and without prior CVD. The quality of the different models was assessed using the Akaike Information Criterion (AIC) [20]. Adding the CCI to the risk model for CVD mortality (adjusted for age, sex and Barthel Index) improved the AIC from 324.2 to 317.7, showing better performance with the CCI included (lower AIC values indicate improved model fit).

Of these four studies, the only study that assessed the incremental value of a multimorbidity variable in relevant sociodemographic subgroups was Livingstone et al., who evaluated calibration and discrimination of sex-specific models by age group [30]. No studies assessed incremental value in ethnic subgroups. In the remaining ten studies, the added incremental value of the multimorbidity variable could not be assessed, as this was not the purpose of the studies. For instance, five studies focused on head-to-head comparisons with other risk scores [22–24, 28, 29], three of which focused on all-cause mortality [22–24]. The remaining studies either assessed the incremental value of another variable in the risk model, evaluated the predictive performance of multiple risk factors simultaneously, or did not include any comparison model.

### Reported limitations of including multimorbidity variables

Limitations regarding the derivation of the multimorbidity variable were identified in the studies. For variables based on claims-based or administrative data, issues such as data quality, missing data, lack of clinical parameter information, and limited knowledge of disease severity were noted [32, 33]. Concerns were also raised about the underestimation of condition prevalence through clinical history assessment [20] and a shorter look-back period [29]. 

The use of diagnostic and pharmacy-based multimorbidity indices also had limitations. Erickson et al. pointed out that using a summary measure with a predefined list of conditions prevents the study from examining the individual conditions’ contributions to study outcomes and fails to detect associations with nonincluded conditions [33]. Different weightings of the indices were derived to predict specific outcomes, and Zhan et al. found that including individual comorbidities in the model performed better than using a weighted score [33]. Furthermore, other predictors, such as frailty, not captured in multimorbidity variables, may improve CVD prediction in competing risk models [30]. Concerns were also noted by Bahrmann et al. on the applicability of the indices in clinical practice and the paucity of evidence for the use of CCI in clinical decision-making processes [20]. 

The use of polypharmacy as a variable was noted as complex by van Bussel, as it is influenced by patient characteristics, preferences, and physician management, and its mechanism of modifying CVD risk remains unclear [31]. In the primary care cohort assessed by van Bussel, a sensitivity analysis defining polypharmacy as ≥ 5 medicine classes excluding cardiovascular medication, polypharmacy was no longer significantly associated with the outcome.

## Discussion

Fourteen studies reported on multivariable prognostic CVD models that included a multimorbidity variable. Of these, three studies specifically looked at the added benefit of a multimorbidity variable in a CVD risk model [20, 21, 30], with a fourth study assessing this as a secondary outcome (primary outcomes of Zhan et al. were overall death and overall hospitalisation) [33]. Only Livingstone et al.’s study looked at model performance by multimorbidity and age subgroups. The challenges of using a summary multimorbidity variable have been described, related to the difficulty in capturing the complexity of how multiple conditions interact at the individual level into a single measure and the reliance on administrative data that can exclude important clinical risk factors and information about disease severity.

All the studies included in this review used weighted multimorbidity measures in CVD risk models. This approach aligns with the findings of a Delphi consensus study on multimorbidity definition and measurement, which confirmed that weighted measures were preferred or considered acceptable for assessing disease severity and predicting outcomes [35]. While other weighting methods exist, condition weights are typically derived using regression coefficients from a multivariable regression model to predict one type of outcome while including age, gender, and existing conditions (or medication classes if pharmaceutical-based) as independent variables [36]. The weights can then be summed to obtain a single numeric score for a particular patient as a summary measure of multimorbidity, accounting for the different impact each condition (or group of conditions or class of medications) has on the outcome of interest [14]. Numerous weighted indices have been derived for various health and multimorbidity-related outcomes [37]. As the CCI and its adaptations are some of the most commonly used weighted multimorbidity indices [38, 39], it was unsurprising that the CCI was used in most of the included studies (12 of the 14 studies) [20–28, 30, 32, 33]. 

Of note, the CCI tool itself has multiple versions and updates. Only five studies reported the version [20, 21, 28, 30, 33], with one study using the original 1987 weightings [20]. The original weightings of the CCI from 1987 were developed to predict one-year mortality in hospitalised patients with comorbidities and may not be as applicable to other populations or outcomes [40]. Additionally, since the CCI was developed in 1987, there have been advances in medical care and changes in the prevalence of specific comorbidities, which may affect the relevance of the original weightings, and many studies have either updated the index or proposed alternative multimorbidity indices to more accurately reflect the impact of some comorbidities on mortality risk in contemporary populations [41]. While these adaptations may still be referred to as the CCI, they have often had modifications to the predefined condition list or condition weightings/outcomes and are, therefore, distinct indices, e.g., the Deyo version of the CCI [39]. Many published studies do not indicate which version, ICD codes or weightings have been used, as was the case for seven studies included in this scoping review [42]. 

### Key evidence gaps identified

The paucity of evidence in clinically relevant populations.

This scoping review revealed that few studies have assessed the incremental value of multimorbidity variables in CVD risk models in primary prevention and secondary prevention cohorts. This knowledge gap may limit our ability to accurately predict cardiovascular disease risk in subpopulations disproportionately impacted by multimorbidity. The type of cohort is highly relevant in prognostic studies, as participants should be at a common point in the progression of the disease/condition [43]. CVD risk prediction models are traditionally developed in cohorts free of CVD at baseline or specific secondary prevention cohorts with a defined follow-up period from diagnosis (e.g., patients one-month post-acute coronary syndrome are different from patients > one-year post-acute coronary syndrome), as this reflects the different prognoses of the groups with respect to atherosclerotic CVD outcomes and the differences in CVD management guidelines. For CVD prognostic studies in which patients are not at a similar point in the disease process, such as those that have used mixed primary and secondary prevention populations, it is unclear to which patients the study findings would be relevant, limiting usefulness in clinical practice [43]. 

Evaluation of the added benefit of multimorbidity in CVD risk models in ethnically diverse populations.

There was a notable absence of research evaluating the addition of multimorbidity variables to CVD risk models with a focus on ethnic subgroup analysis. While Livingstone et al. evaluated the incremental value of the CCI in CVD risk prediction models in a primary care cohort, it is important to note that their study was conducted in a predominantly European/White population (> 90%). The generalisability of the findings to more ethnically diverse populations is uncertain, as disparities in the incidence of CVD, chronic disease prevalence and the distinct multimorbidity profiles seen across ethnic groups [44–46] could affect the performance of the model. Moreover, there is evidence that some clinical CVD risk prediction models may not perform as well in non-European populations [47, 48]. Evaluation of clinical CVD risk prediction models that incorporate multimorbidity variables across ethnic subgroups is important for understanding model performance in different ethnic groups, assessing the utility and applicability of the risk model in more diverse populations, and ensuring the use of the risk model is likely to contribute to more equitable health outcomes.

The optimal measurement of the cumulative effect of multiple conditions.

There is a lack of literature evaluating the incremental value of alternative multimorbidity variables beyond the CCI (e.g., alternative diagnosis-based multimorbidity variables with different predefined condition lists or pharmaceutical-based multimorbidity variables). Comparing the performance of alternative multimorbidity variables with different predefined condition lists or pharmaceutical-based multimorbidity variables could be helpful for future research, particularly given that many multimorbidity-weighted indices have been derived and validated for non-CVD outcomes. In addition, Erickson et al. highlighted that assessing a multimorbidity summary variable in CVD risk models does not provide insight into which conditions are most significant and how they interact within multimorbidity patterns [21]. Multimorbidity summary variables do not capture the complexity and nuances of the individual diseases and their interactions, as different combinations of diseases/comorbidities may have different impacts on a patient’s risk of cardiovascular disease.

### Limitations

This scoping review was designed to be broad. It included a well-defined research question, a comprehensive search strategy with multiple databases, clear and transparent screening and data extraction processes, and an analysis of relevant gaps in the literature. While this scoping review facilitated a broad exploration of the topic, it is important to note that no formal assessment of study quality was conducted. This is consistent with the scoping review methodology, which typically does not include such assessments. This scoping review did not specifically explore the relevance of competing risk methodologies in patients with multimorbidity. While this enabled the review to focus on one specific research question, other factors that may impact the accuracy of CVD risk prediction in adults with multimorbidity were not addressed, such as the higher occurrence of non-CVD death. Any use of competing risk models was noted in Table 1 if reported in the study. Other limitations included the search strategy’s reliance on selected keywords, which might have overlooked less commonly reported multimorbidity measures used in CVD risk models, the review’s exclusion of non-English publications, studies predating 2012, and the heterogeneous nature of included studies. Furthermore, all screening was conducted solely by the lead author, which could introduce bias. However, the review captured a wide range of relevant information to identify gaps in the literature and inform the development of future research questions.

## Conclusion

This methodological scoping review provides a comprehensive analysis of the incorporation of multimorbidity variables in CVD risk prediction models, focusing on assessing their incremental value in primary and secondary prevention cohorts. Despite the importance of this topic, the review uncovered several notable gaps in the existing literature, including limited research, insufficient evaluation in diverse populations, and a lack of comparison between alternative multimorbidity variables. Addressing these gaps is crucial for ensuring that CVD risk assessment and management are appropriate for individuals with pre-existing long-term conditions and that any associated inequities are addressed. Future research is needed to investigate the benefits of using multimorbidity variables in cardiovascular disease risk prediction models in diverse populations, with appropriate ethnic and multimorbidity subgroup analysis, to support the development of more effective evidence-based strategies for people with multimorbidity.

## Electronic supplementary material

Below is the link to the electronic supplementary material.

Supplementary Material 1: Additional File 1

Supplementary Material 2: Additional File 2

Supplementary Material 3: Additional File 3

## Data Availability

All data generated or analysed during this study are included in this published article [and its supplementary information files].

## Abbreviations

ACS: Acute coronary syndrome
adjHR: Adjusted hazard ratio
adjOR: Adjusted odds ratio
AIC: Akaike Information Criterion
AUC: Area under the Receiver Operating Characteristic (ROC) Curve
CCI: Charlson Comorbidity Index
CDS: Chronic Disease Score
COPD: Chronic obstructive pulmonary disease
CVD: Cardiovascular disease
GRPI: Grace Risk Prediction Index
HIV/AIDS: Human immunodeficiency virus infection and acquired immune deficiency syndrome
ICD: International Classification of Diseases
IPCW: Inverse probability of censoring weighting
IQR: Interquartile range
MM: Multimorbidity
NNT: Number needed to treat
NRI: Net reclassification improvement
PBDI: Pharmacy–Based Disease Index
PRISMA: Preferred Reporting Items for Systematic Reviews and Meta–Analyses
PRISMA: ScR–Preferred Reporting Items for Systematic Reviews and Meta–Analyses extension for Scoping Reviews
SD: Standard deviation
SHR: Subdistribution hazard ratio
UK: United Kingdom
US: United States

## References

[1] Roth GA, Mensah GA, Johnson CO, Addolorato G, Ammirati E, Baddour LM, et al. Global burden of cardiovascular diseases and risk factors, 1990–2019: update from the GBD 2019 study. J Am Coll Cardiol. 2020;76(25):2982–3021. 33309175 10.1016/j.jacc.2020.11.010 PMC7755038

[2] Barnett K, Mercer SW, Norbury M, Watt G, Wyke S, Guthrie B. Epidemiology of Multimorbidity and implications for health care, research, and medical education: A cross-sectional study. Lancet. 2012;380(9836):37–43. 22579043 10.1016/S0140-6736(12)60240-2

[3] Tran J, Norton R, Conrad N, Rahimian F, Canoy D, Nazarzadeh M, Rahimi K. Patterns and Temporal trends of comorbidity among adult patients with incident cardiovascular disease in the UK between 2000 and 2014: A population-based cohort study. PLoS Med. 2018;15(3):e1002513. 29509757 10.1371/journal.pmed.1002513 PMC5839540

[4] Huang Y, Kung P, Chiu L, Tsai W. Related factors and incidence risk of acute myocardial infarction among the people with disability: A National population-based study. Res Dev Disabil. 2015;36 C:366–75. 25462496 10.1016/j.ridd.2014.10.019

[5] Kang EJ, Lee YG, Koo M, Lee K, Park IH, Kim JS, Choi YJ. The risk of cardiovascular disease and stroke in survivors of head and neck cancer in Korea. Health Sci Rep. 2022;5(2):e517. 35224218 10.1002/hsr2.517 PMC8855631

[6] Mamas MA, Fath-Ordoubadi F, Danzi GB, Spaepen E, Kwok CS, Buchan I, et al. Prevalence and impact of co-morbidity burden as defined by the Charlson co-morbidity index on 30-day and 1- and 5-year outcomes after coronary stent implantation (from the Nobori-2 study). Am J Cardiol. 2015;116(3):364–71. 26037294 10.1016/j.amjcard.2015.04.047

[7] Canivell S, Muller O, Gencer B, Heg D, Klingenberg R, Räber L, et al. Prognosis of cardiovascular and non-cardiovascular Multimorbidity after acute coronary syndrome. PLoS ONE. 2018;13(4):e0195174. 29649323 10.1371/journal.pone.0195174 PMC5896917

[8] Okkonen M, Havulinna AS, Ukkola O, Huikuri H, Pietilä A, Koukkunen H, et al. Risk factors for major adverse cardiovascular events after the first acute coronary syndrome. Ann Med. 2021;53(1):817–23. 34080496 10.1080/07853890.2021.1924395 PMC8183550

[9] Tonelli M, Wiebe N, Guthrie B, James MT, Quan H, Fortin M, et al. Comorbidity as a driver of adverse outcomes in people with chronic kidney disease. Kidney Int. 2015;88(4):859–66. 26221754 10.1038/ki.2015.228

[10] Badawy MAEMD, Naing L, Johar S, Ong S, Rahman HA, Tengah DSNAP, et al. Evaluation of cardiovascular diseases risk calculators for CVDs prevention and management: scoping review. BMC Public Health. 2022;22(1):1742. 36104666 10.1186/s12889-022-13944-w PMC9471025

[11] Nguyen QD, Odden MC, Peralta CA, Kim DH. Predicting risk of atherosclerotic cardiovascular disease using pooled cohort equations in older adults with frailty, Multimorbidity, and competing risks. J Am Heart Assoc. 2020;9(18):e016003. 32875939 10.1161/JAHA.119.016003 PMC7727000

[12] Hippisley-Cox J, Coupland C, Brindle P. Development and validation of QRISK3 risk prediction algorithms to estimate future risk of cardiovascular disease: prospective cohort study. BMJ. 2017;357. 10.1136/bmj.j2099 PMC5441081 28536104

[13] Livingstone S, Morales DR, Donnan PT, Payne K, Thompson AJ, Youn JH, Guthrie B. Effect of competing mortality risks on predictive performance of the QRISK3 cardiovascular risk prediction tool in older people and those with comorbidity: external validation population cohort study. Lancet Healthy Longev. 2021;2(6):e352–61. 34100008 10.1016/S2666-7568(21)00088-X PMC8175241

[14] Sarfati D. Review of methods used to measure comorbidity in cancer populations: no gold standard exists. J Clin Epidemiol. 2012;65(9):924–33. 22739245 10.1016/j.jclinepi.2012.02.017

[15] Tricco AC, Lillie E, Zarin W, O’Brien K, Colquhoun H, Kastner M, et al. A scoping review on the conduct and reporting of scoping reviews. BMC Med Res Methodol. 2016;16(1):15. 26857112 10.1186/s12874-016-0116-4 PMC4746911

[16] Arksey H, O’Malley L. Scoping studies: towards a methodological framework. Int J Soc Res Methodol. 2005;8(1):19–32.

[17] Tricco AC, Lillie E, Zarin W, O’Brien KK, Colquhoun H, Levac D, et al. PRISMA extension for scoping reviews (PRISMA-ScR): checklist and explanation. Ann Intern Med. 2018;169(7):467–73. 30178033 10.7326/M18-0850

[18] Peters MD, Godfrey CM, Khalil H, McInerney P, Parker D, Soares CB. Guidance for conducting systematic scoping reviews. JBI Evid Implement. 2015;13(3):141–6. 10.1097/XEB.0000000000000050 26134548

[19] Moher D, Shamseer L, Clarke M, et al. Preferred reporting items for systematic review and meta-analysis protocols (PRISMA-P) 2015 statement. Syst Reviews. 2015;4(1):1–9. 10.1186/2046-4053-4-1 PMC4320440 25554246

[20] Bahrmann A, Benner L, Christ M, Bertsch T, Sieber CC, Katus H, Bahrmann P. The Charlson comorbidity and Barthel index predict length of hospital stay, mortality, cardiovascular mortality and rehospitalization in unselected older patients admitted to the emergency department. Aging Clin Exp Res. 2019;31(9):1233–42. 30406920 10.1007/s40520-018-1067-x

[21] Erickson SR, Cole E, Kline-Rogers E, Eagle KA. The addition of the Charlson comorbidity index to the GRACE risk prediction index improves prediction of outcomes in acute coronary syndrome. Popul Health Manag. 2014;17(1):54–9. 23965044 10.1089/pop.2012.0117

[22] Fauchier L, Bisson A, Bodin A, Clementy N, Pierre B, Angoulvant D et al. Predicting mortality and mode of death by clinical score systems for patients with atrial fibrillation. Eur Heart J. 2019; Conference:European.

[23] Fauchier L, Bisson A, Bodin A, Herbert J, Clementy N, Pierre B et al. Prediction of mortality and mode of death by clinical risk score systems in 2.6 million patients with atrial fibrillation: A nationwide analysis. European Heart Journal. Conference: European Society of Cardiology Congress, ESC 2020.Virtual.

[24] Fauchier L, Bodin A, Bentounes SA, Bisson A, Herbert J, Genet T et al. Prediction of mortality and mode of death by multimorbidity and clinical risk score systems in patients with heart failure: A nationwide analysis. European Journal of Heart Failure. 2022; Conference:Heart.

[25] Gelow JM, Mudd JO, Chien CV, Lee CS. Usefulness of cognitive dysfunction in heart failure to predict cardiovascular risk at 180 days. Am J Cardiol. 2015;115(6):778–82. 25644853 10.1016/j.amjcard.2014.12.040 PMC4353403

[26] Haji K, Marwick TH, Stewart S, Carrington M, Chan YK, Chan W, et al. Incremental value of global longitudinal strain in the long-term prediction of heart failure among patients with coronary artery disease. J Am Soc Echocardiogr. 2022;35(2):187–95. 34508839 10.1016/j.echo.2021.09.003

[27] Jeong S, Lee G, Choi S, Kim KH, Chang J, Kim SM, et al. Estimating risk of cardiovascular disease among long-term colorectal cancer survivors: A nationwide cohort study. Front Cardiovasc Med. 2021;8:721107. 35111822 10.3389/fcvm.2021.721107 PMC8801493

[28] Kayama K, Yamada T, Morita T, Furukawa Y, Tamaki S, Iwasaki Y et al. Prognostic impact of AHEAD risk score in patients with acute decompensated heart failure: A prospective comparative study with the age-adjusted Charlson comorbidity index. Eur Heart J. 2018; Conference:European.

[29] Lip GYH, Genaidy A, Tran G, Marroquin P, Estes C, Sloop S. Improving stroke risk prediction in the general population: A comparative assessment of common clinical rules, a new multimorbid index, and machine-learning-based algorithms. Thromb Haemost. 2022;122(1):142–50. 33765685 10.1055/a-1467-2993

[30] Livingstone SJ, Guthrie B, Donnan PT, Thompson A, Morales DR. Predictive performance of a competing risk cardiovascular prediction tool CRISK compared to QRISK3 in older people and those with comorbidity: population cohort study. BMC Med. 2022;20(1):152. 35505353 10.1186/s12916-022-02349-6 PMC9066924

[31] van Bussel EF, Richard E, Busschers WB, Steyerberg EW, van Gool WA, van Moll EP, Hoevenaar-Blom MP. A cardiovascular risk prediction model for older people: development and validation in a primary care population. J Clin Hypertens. 2019;21(8):1145–52. 10.1111/jch.13617 PMC6772108 31294917

[32] Walkey AJ, Knox DB, Myers LC, Thai KK, Jacobs JR, Kipnis P, et al. Prognostic accuracy of presepsis and intrasepsis characteristics for prediction of cardiovascular events after a sepsis hospitalization. Crit Care Explor. 2022;4(4):e0674. 35425904 10.1097/CCE.0000000000000674 PMC9000037

[33] Zhan ZW, Chen YA, Dong YH. Comparative performance of comorbidity measures in predicting health outcomes in patients with chronic obstructive pulmonary disease. Int J COPD. 2020;15:335–44. 10.2147/COPD.S229646 PMC7024789 32103932

[34] Fox KA, Dabbous OH, Goldberg RJ, Pieper KS, Van de Eagle KA, et al. Prediction of risk of death and myocardial infarction in the six months after presentation with acute coronary syndrome: prospective multinational observational study (GRACE). BMJ. 2006;333(7578):1091. 17032691 10.1136/bmj.38985.646481.55 PMC1661748

[35] Ho IS, Azcoaga-Lorenzo A, Akbari A, Davies J, Kamlesh K, Kadam UT, et al. Measuring Multimorbidity in research: Delphi consensus study. BMJ Med. 2022;1:e000247. 36936594 10.1136/bmjmed-2022-000247 PMC9978673

[36] Mehta HB, Mehta V, Girman CJ, Adhikari D, Johnson ML. Regression coefficient–based scoring system should be used to assign weights to the risk index. J Clin Epidemiol. 2016;79:22–8. 27181564 10.1016/j.jclinepi.2016.03.031

[37] Lee ES, Koh HL, Ho EQ, Teo SH, Wong FY, Ryan BL, et al. Systematic review on the instruments used for measuring the association of the level of Multimorbidity and clinically important outcomes. BMJ Open. 2021;11(5):e041219. 33952533 10.1136/bmjopen-2020-041219 PMC8103380

[38] Yurkovich M, Avina-Zubieta JA, Thomas J, Gorenchtein M, Lacaille D. A systematic review identifies valid comorbidity indices derived from administrative health data. J Clin Epidemiol. 2015;68(1):3–14. 25441702 10.1016/j.jclinepi.2014.09.010

[39] Charlson ME, Carrozzino D, Guidi J, Patierno C. Charlson comorbidity index: A critical review of clinimetric properties. Psychother Psychosom. 2022;91(1):8–35. 34991091 10.1159/000521288

[40] Charlson ME, Pompei P, Ales KL, MacKenzie CR. A new method of classifying prognostic comorbidity in longitudinal studies: development and validation. J Chronic Dis. 1987;40(5):373–83. 3558716 10.1016/0021-9681(87)90171-8

[41] Quan H, Li B, Couris CM, Fushimi K, Graham P, Hider P, et al. Updating and validating the Charlson comorbidity index and score for risk adjustment in hospital discharge abstracts using data from 6 countries. Am J Epidemiol. 2011;173(6):676–82. 21330339 10.1093/aje/kwq433

[42] Brusselaers N, Lagergren J. The Charlson comorbidity index in registry-based research. Methods Inf Med. 2017;56(5):401–6. 29582935 10.3414/ME17-01-0051

[43] Fineout-Overholt E, Melnyk BM. Evaluation of studies of prognosis. Evid Based Nurs. 2004;7(1):4–8. 14994681 10.1136/ebn.7.1.4

[44] Chan WC, Wright C, Riddell T, Wells S, Gala G, Jackson R. Ethnic and socioeconomic disparities in the prevalence of cardiovascular disease in new Zealand. N Z Med J. 2008;121(1285):11–20. 19079433

[45] Ng Fat L, Patil P, Mindell JS, Manikam L, Scholes S. Ethnic differences in Multimorbidity after accounting for social-economic factors, findings from the health survey for England. Eur J Public Health. 2023;33(6):959–67. 37634091 10.1093/eurpub/ckad146 PMC10710338

[46] Stanley J, Semper K, Millar E, Sarfati D. Epidemiology of Multimorbidity in new Zealand: A cross-sectional study using national-level hospital and pharmaceutical data. BMJ Open. 2018;8(5):e021689. 29794103 10.1136/bmjopen-2018-021689 PMC5988147

[47] Kist JM, Vos RC, Mairuhu ATA, Struijs JN, van Peet PG, Vos HMM et al. SCORE2 cardiovascular risk prediction models in an ethnic and socioeconomic diverse population in the Netherlands: an external validation study. eClinicalMedicine. 2023;57. 10.1016/j.eclinm.2023.101862 PMC9971516 36864978

[48] Kasim SS, Ibrahim N, Malek S, Ibrahim KS, Aziz MF, Song C et al. Validation of the general Framingham risk score (FRS), SCORE2, revised PCE and WHO CVD risk scores in an Asian population. Lancet Reg Health– Western Pac. 2023;35. 10.1016/j.lanwpc.2023.100742 PMC10326687 37424687

