# Supplementary material for: Scoping review of the use of multimorbidity variables in cardiovascular disease risk prediction
Source: BMC Public Health. 2025 Mar 17;25:1027. doi: 10.1186/s12889-025-22169-6 (PMC11912685; doi:10.1186/s12889-025-22169-6)
Supplement: Supplementary file 2 — Supplementary Material 2: Additional File 2 [file 12889_2025_22169_MOESM2_ESM.pdf]

Additional File 2

Search strategy

Table 1 Embase 1980 to September 21, 2022

#	Searches	Results
1	(((co?morbid* or multi?morbid*) adj3 (count* or measure* or weighted or score* or algorithm* or composite* or instrument* or indice* or index or cluster analys* or factor analys* or latent class analys*)) or (disease count* or drug count* or pharmaceutical count* or charlson index or charlson comorbidity index or CCI or Elixhauser comorbidity index or Elixhauser index or Elder? risk assessment or elderly risk assessment or multi?morbidity?weighted index or MWI or rxrisk or chronic disease score or case?mix or adjusted clinical group* or ACG or cumulative illness rating scale or CIRS or P3 or Pharmaceutical Prescribing Profile or M3 or DRG or Diagnosis?Related Group* or polypharmacy)).mp.	174,233
2	exp Charlson Comorbidity Index/ or exp comorbidity assessment/ or exp Elixhauser comorbidity index/	36,047
3	((cardiovascular or CVD or IHD or CHD or coronary or stroke or cerebrovascular disease* or vascular disease* or angina or heart disease* or cardiac event* or myocardial infarction or acute coronary syndrome* or ACS or coronary artery disease* or CAD or heart failure) adj2 (risk* or predict*)).mp.	415,532
4	cardiovascular disease/ or heart disease/ or vascular disease/ or peripheral vascular disease/ or cerebrovascular disease/ or coronary artery disease/ or cardiovascular mortality/ or heart failure/	975,421

5	mortality risk/ or recurrence risk/ or patient risk/ or risk algorithm/ or risk/ or risk assessment/ or mortality risk score/ or population risk/ or prediction/	1,691,584
6	exp cardiovascular risk/ or exp coronary risk/ or exp Framingham risk score/	241,318
7	1 or 2	176,362
8	4 and 5	147,431
9	3 or 6 or 8	505,387
10	7 and 9	5,545
11	Limits (English language and year 2012 - Current)	4,500

Notes: mp=title, abstract, heading word, drug trade name, original title, device manufacturer, drug manufacturer, device trade name, keyword heading word, floating subheading word, candidate term word

Table 2 Ovid MEDLINE(R) and Epub Ahead of Print, In-Process, In-Data-Review & Other Non-Indexed Citations, Daily and Versions(R) 1946 to September 23, 2022

#	Searches	Results
1	(((co?morbid* or multi?morbid*) adj3 (count* or measure* or weighted or score* or algorithm* or composite* or instrument* or indice* or index or cluster analys* or factor analys* or latent class analys*)) or (disease count* or drug count* or pharmaceutical count* or charlson index or charlson comorbidity index or CCI or Elixhauser comorbidity index or Elixhauser index or Elder? risk assessment or elderly risk assessment or multi?morbidity?weighted index or MWI or rxrisk or chronic disease score or case?mix or adjusted clinical group* or ACG or cumulative illness rating scale or CIRS or P3 or Pharmaceutical Prescribing Profile or M3 or DRG or Diagnosis?Related Group* or polypharmacy)).mp	113,182
2	((cardiovascular or CVD or IHD or CHD or coronary or stroke or cerebrovascular disease* or vascular disease* or angina or heart disease* or cardiac event* or	194,555

	myocardial infarction or acute coronary syndrome* or ACS or coronary artery disease* or CAD or heart failure) adj2 (risk* or predict*)).mp	
3	vascular diseases/ or cerebrovascular disorders/ or myocardial ischemia/ or acute coronary syndrome/ or angina pectoris/ or coronary disease/ or myocardial infarction/ or stroke/ or peripheral vascular diseases/ or cardiovascular diseases/ or heart diseases/ or heart failure/	892,839
4	risk/ or risk adjustment/ or risk assessment/ or exp survival analysis/ or exp regression analysis/	1,056,547
5	exp cardiovascular risk/ or exp heart disease risk factors/	4,902
6	3 and 4	99,470
7	2 or 5 or 6	262,758
8	1 and 7	1,871
9	Limits (English language and year 2012 - Current)	1,340

Notes: mp=title, abstract, heading word, drug trade name, original title, device manufacturer, drug manufacturer, device trade name, keyword heading word, floating subheading word, candidate term word

Table 3 Cochrane Library publication date from Jan 2012 to September 2022

#	Searches	Results
1	((((co?morbid* or multi?morbid*) near/3 (count* or measure* or weighted or score* or algorithm* or composite* or instrument* or indice* or index or "cluster analys*" or "factor analys*" or "latent class analys*")) or ("disease count*" or "drug count*" or "pharmaceutical count*" or "charlson index" or "charlson comorbidity index" or "CCI" or "Elixhauser comorbidity index" or "Elixhauser index" or "Elder? risk assessment" or "elderly risk assessment" or "multi?morbidity?weighted index" or MWI or rxrisk or "chronic disease score"	8,728

	or "case?mix" or "adjusted clinical group*" or ACG or "cumulative illness rating scale" or CIRS or P3 or "Pharmaceutical Prescribing Profile" or M3 or DRG or "Diagnosis?Related Group*" or polypharmacy))):ti,ab,kw	
2	MeSH descriptor: [Multimorbidity] explode all trees or MeSH descriptor: [Comorbidity] explode all trees	3,847
3	((cardiovascular or CVD or IHD or CHD or coronary or stroke or "cerebrovascular disease*" or "vascular disease*" or angina or "heart disease*" or "cardiac event*" or "myocardial infarction" or "acute coronary syndrome*" or ACS or "coronary artery disease*" or CAD or "heart failure") near/2 (risk* or predict*)):ti,ab,kw	33,295
4	(MeSH descriptor: [Cardiovascular Diseases] this term only) or (MeSH descriptor: [Heart Diseases] this term only) or (MeSH descriptor: [Vascular Diseases] this term only) or (MeSH descriptor: [Heart Failure] explode all trees) or (MeSH descriptor: [Myocardial Ischemia] explode all trees) or (MeSH descriptor: [Peripheral Vascular Diseases] explode all trees) or (MeSH descriptor: [Cerebrovascular Disorders] explode all trees) or (MeSH descriptor: [Coronary Disease] explode all trees) or (MeSH descriptor: [Stroke] explode all trees)	68,182
5	MeSH descriptor: [Risk] explode all trees	39,608
6	MeSH descriptor: [Heart Disease Risk Factors] explode all trees	314
7	1 or 2	12,390
8	4 and 5	11,437
9	3 or 6 or 8	39,834
10	7 and 9	734
11	Limits (year 2012 - Current)	508
