# Supplementary material for: Scoping review of the use of multimorbidity variables in cardiovascular disease risk prediction
Source: BMC Public Health. 2025 Mar 17;25:1027. doi: 10.1186/s12889-025-22169-6 (PMC11912685; doi:10.1186/s12889-025-22169-6)
Supplement: Supplementary file 3 — Supplementary Material 3: Additional File 3 [file 12889_2025_22169_MOESM3_ESM.pdf]

Additional File 3

Data Charting Form

Data chart heading	Description
**Author**	Name of author/s
**Date**	Date article sourced
**Title of study**	Title of the article or study
**Publication year**	The year that the article was published
**Publication type**	Journal, website, conference, etc.
**Study details and design (if applicable)**	Type of study, empirical or review, etc.
**Keywords**	What keywords were present
**Study sector/setting**	Country/state/hospital/mortuary
**Study population**	Population studied with regard to demographics
**Prevention cohort type**	Detail whether primary prevention cohort, secondary prevention cohort or mixed cohort
**Multimorbidity measure**	Definition and measures of multimorbidity presented in the study, including the number of diseases, lookback period and version
**Outcome**	List all outcomes
**Time horizon/Follow-up**	Period of time
**Number of events**	Reported number of first cardiovascular events in the cohort
**Types of data sources included**	Detail the data sources
**Type of risk prediction model**	Type of regression model (e.g., Cox proportional hazards regression, competing risk regression)
**Models compared**	Describe the models compared
**Adjusted hazard ratio of multimorbidity variable**	Result reported in fully adjusted model
**Performance metrics used**	List the performance metrics used and the results reported
**Assessment of adding multimorbidity variable to the CVD risk model (assessed at all or assessed sufficiently)**	Incremental value of multimorbidity variable assessed (compared to reference group of established predictors without multimorbidity variable) and appropriate metrics used
**Reported challenges or limitations of including the multimorbidity variable**	Challenges or limitations reported with respect to applying a multimorbidity variable
**Conclusion**	Important aspects of the conclusion
**Most significant findings**	Noteworthy results of the study
**Most relevant findings**	Findings that contribute to the research question
